# Association between autoimmune disease and colorectal cancer: a retrospective case-control study of 120 876 patients

**DOI:** 10.1136/bmjgast-2025-001886

**Published:** 2025-10-05

**Authors:** Sven Heiko Loosen, Frederik Hansen, Tom Luedde, Christoph Roderburg, Karel Kostev

**Affiliations:** 1 University Hospital, Department of Gastroenterology, Hepatology and Infectious Diseases, Heinrich-Heine-Universitat Dusseldorf, Düsseldorf, Germany; 2 Gastroenterology, Hepatology and Infectious Disease, Heinrich-Heine-Universität Düsseldorf, Düsseldorf, Germany; 3 IQVIA Germany, Frankfurt, Germany

**Keywords:** colorectal cancer, autoimmune disease, INFLAMMATORY BOWEL DISEASE

## Abstract

**Objective:**

Colorectal cancer (CRC) is one of the most frequently diagnosed cancers and a leading cause of cancer-related deaths worldwide. While inflammatory bowel disease (IBD) is a well-established risk factor for CRC, the potential link between other autoimmune diseases and CRC is unclear. In light of the growing prevalence of autoimmune diseases and their recognised link to various malignancies, this study seeks to investigate whether different autoimmune diseases are associated with CRC.

**Methods:**

A total of 20 146 patients with an initial diagnosis of CRC and 100 730 propensity score-matched cancer-free individuals were identified from the Disease Analyzer database (IQVIA). Univariable conditional logistic regression models were used to examine whether each autoimmune disorder was associated with subsequent CRC diagnosis.

**Results:**

Only IBD was significantly associated with CRC (OR 1.53; 95% CI 1.33 to 1.75). Type 1 diabetes, rheumatic diseases, autoimmune thyroiditis, and multiple sclerosis did not show a significant association with CRC. Psoriasis showed a non-significant trend towards an association with CRC (OR 1.11; 95% CI 0.97 to 1.27). Coeliac disease was not associated with the development of CRC (OR 1.06; 95% CI 0.69 to 1.64). A sex-stratified analysis revealed that the association between IBD and CRC was similar in both women (OR 1.48; 95% CI 1.22 to 1.81) and men (OR 1.57; 95% CI 1.29 to 1.89). No significant sex differences for any other autoimmune disease were observed.

**Conclusion:**

The presence of IBD, but not any other autoimmune diseases, was significantly associated with a subsequent CRC. This finding serves to emphasise the significance of routine screening for patients suffering from IBD.

**WHAT IS ALREADY KNOWN ON THIS TOPIC**

- Colorectal cancer (CRC) is a leading cause of cancer-related death worldwide, with established risk factors including age, family history, hereditary syndromes, inflammatory bowel disease (IBD), and certain lifestyle factors.
- Chronic inflammation, such as that seen in IBD, is a well-known driver of CRC development.
- Autoimmune diseases are increasingly prevalent and have been linked to elevated cancer risks in various organs. However, except for IBD, the association between other autoimmune diseases and CRC remains poorly understood and understudied.

**WHAT THIS STUDY ADDS**

- This study comprehensively examines the association between the 10 most common autoimmune diseases and CRC using a large, representative outpatient database.
- It confirms the well-established link between IBD and CRC but finds no significant association between CRC and other autoimmune diseases such as type 1 diabetes, rheumatoid arthritis, or coeliac disease.

**HOW THIS STUDY MIGHT AFFECT RESEARCH, PRACTICE OR POLICY**

- By showing that only IBD among common autoimmune diseases is linked to CRC, this study helps focus future research on the mechanisms driving cancer risk in IBD.
- Clinically, it reinforces the importance of targeted CRC screening and surveillance in patients with IBD, potentially influencing guidelines and healthcare policies to prioritise resources for this high-risk group while avoiding unnecessary screening in patients with other autoimmune conditions.

## Introduction

 Colorectal cancer (CRC) is the third most commonly diagnosed cancer worldwide and the second leading cause of cancer-related mortality.^1^ The primary treatment depends on the tumour’s location and spread. For non-metastatic colon cancer, surgery is the main treatment, with chemotherapy added in certain cases. Rectal cancer often requires chemoradiotherapy before surgery due to its location and recurrence risk.^2^ In metastatic CRC, systemic therapy, including chemotherapy, targeted agents and immunotherapy, is key, with choices guided by molecular markers such asRAS, BRAF and MSI status.^3^

The major risk factors for CRC include being over 50 years of age, a family history of the disease, hereditary syndromes such as Lynch syndrome, inflammatory bowel disease (IBD) and specific lifestyle factors including a high intake of red and processed meat, obesity, smoking and alcohol consumption.^4^,^6^ These factors have been shown to promote genetic and epigenetic changes that drive cancer development. Early diagnosis of CRC is imperative for a favourable outcome. Colonoscopies for screening purposes have already demonstrated favourable outcomes in this regard; however, they are not universally accepted by patients.^7^ It is therefore important to identify further risk factors in order to improve the outcome of CRC through more targeted screening.

Autoimmune diseases are increasing in prevalence and have been associated with an elevated risk of cancer.^8^ For instance, rheumatoid arthritis (RA) has been linked to an elevated probability of developing lung, cervical and oropharyngeal cancers, as well as haematological malignancies.^9 10^ In a similar vein, patients with systemic lupus erythematosus (SLE) or multiple sclerosis have been shown to have an elevated risk of developing both blood cancers and solid tumours, including lung cancer.^11^,^13^

Numerous studies have confirmed that the presence of IBD increases the risk of CRC.^14 15^ However, the studies on the association between other autoimmune diseases and CRC are rare, making further research required. The objective of the present study is to investigate the potential association between the 10 most frequent autoimmune diseases and CRC.

## Methods

### Database

This study used data from the Disease Analyzer database (IQVIA). Details of this database have been published previously.^16^ In brief, the Disease Analyzer database contains data on demographic variables, diagnoses and prescriptions of outpatients from general practices in Germany. The database covers about 1300 general practices in Germany. The panel of practices included in the Disease Analyzer database has previously been shown to be representative of office-based practices in Germany.^16^

### Study population

**Figure 1:**
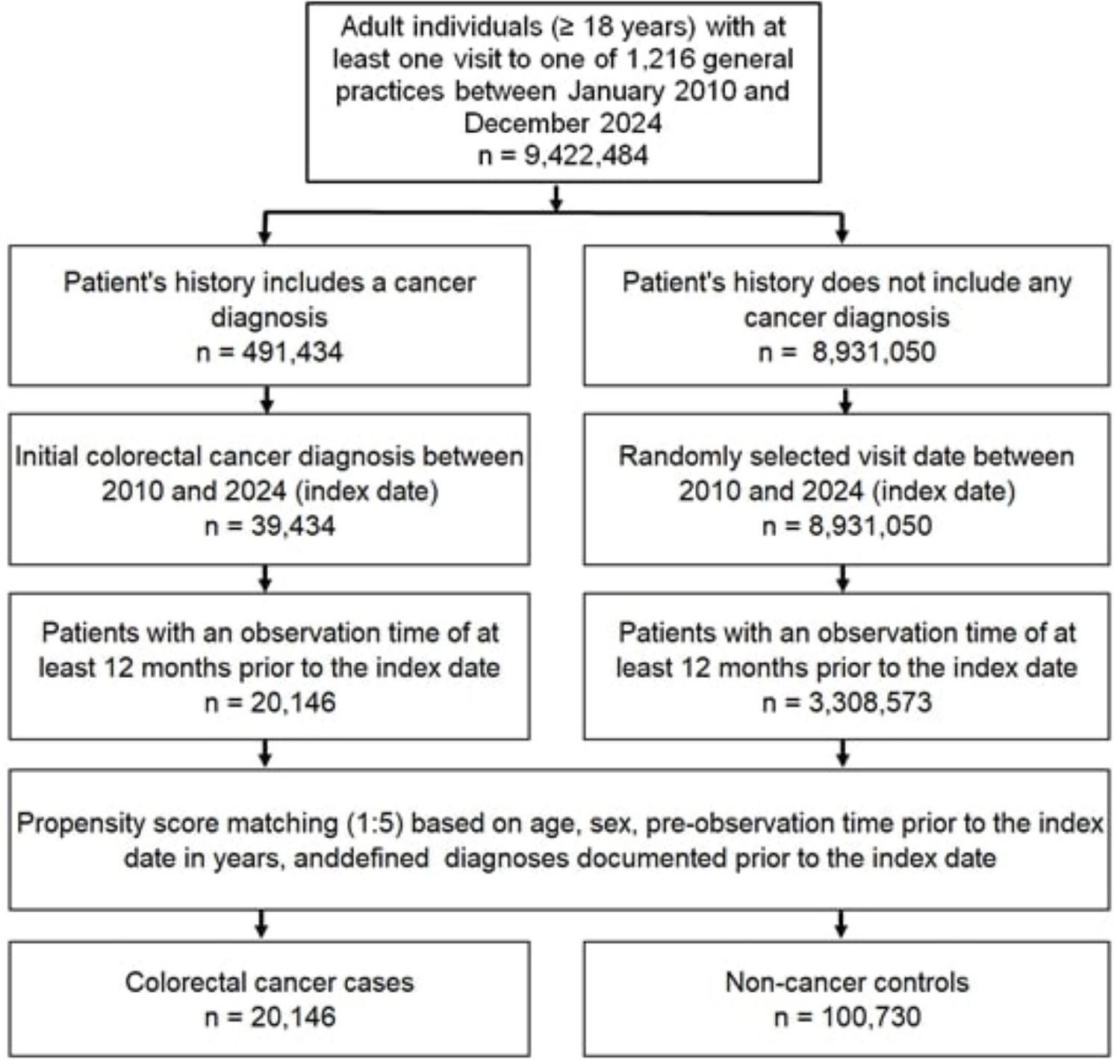
Selection of study patients.

The study population included all patients aged ≥18 years with first diagnosis of CRC (ICD-10 codes: C18-C20) between January 2010 and December 2024 (index date) who had at least 1 year of observation prior to the index date. Controls were individuals with no history of cancer who were matched (5:1) by nearest neighbour propensity scores based on age, sex, year of index date, years of observation before the index date, and diagnoses documented before the index date including type 2 diabetes mellitus (T2D) (ICD-10: E11), obesity (ICD-10: E66), nicotine dependence (ICD-10: F17), chronic obstructive pulmonary disease (COPD) (ICD-10: J44), and colorectal polyps (ICD-10: K85, K86). T2D, obesity, polyps, and tobacco use are considered risk factors for CRC. As information on tobacco use is not available, we used diagnoses of nicotine dependence and COPD which may proxy for smoking behaviour. For individuals without cancer (controls), the index date was a randomly selected visit date between January 2010 and December 2024. The flowchart of study participants is shown in figure 1. For propensity score matching, a standardised mean difference (SMD) of less than 0.1 was allowed, indicating adequate covariate adjustment. The STROBE checklist for this report is provided in the online supplemental material.

### Study outcome

The outcome of the study was the association between autoimmune diseases documented in the complete patient history before the index date and subsequent diagnosis of CRC. The diagnoses of interest were type 1 diabetes (T1D) (ICD-10: I10), IBD (ICD-10: K50, K51), RA (ICD-10: M05, M06), psoriasis (ICD-10: L40), SLE (ICD-10: M32), autoimmune thyroiditis (ICD-10: E06.3), Grave’s disease (ICD-10: E05.0), multiple sclerosis (ICD-10: G35), ankylosing spondylitis (ICD-10: M45) and coeliac disease (ICD-10: K90.0).

### Statistical analyses

Univariable conditional logistic regression models and estimated ORs with 95% CIs were used to examine whether each autoimmune disorder was associated with subsequent CRC diagnoses. This model was also calculated separately for female and male patients. A p value <0.05 was considered statistically significant. All analyses were performed using SAS V.9.4 (SAS Institute, Cary, North Carolina, USA).

## Results

### Baseline characteristics

Following a 1:5 matching process, the study incorporated 20 146 CRC cases and 100 730 controls without cancer. The mean age at the index date was 69.7 years for patients with CRC and 70.0 years for controls. The sex distribution was similar between groups, with females comprising 47% of each cohort. There was no significant difference in the prevalence of diabetes mellitus or obesity between the groups. However, individuals diagnosed with CRC exhibited marginally elevated rates of COPD and colorectal polyps. The mean pre-observation period for both groups was 8.6 years prior to the index date. Table 1 presents the prevalence of the most common predefined co-diagnoses.

**Table 1:** Characteristics of study patients after 1:5 matching

Variable	Colorectal cancer (n=20 146)	No cancer (n=100 730)	SMD	P value
Age (in years)				
Mean (SD)	69.7 (13.0)	70.0 (13.0)	−0.021	0.038
*≤*60	4703 (23.3)	22 999 (22.8)		0.052
61–70	4682 (23.2)	23 096 (22.9)		
71–80	6315 (31.4)	31 486 (31.3)		
>80	4446 (22.1)	23 149 (23.0)		
Sex				
Female	9483 (47.1)	47 241 (46.9)	0.002	0.654
Male	10.663 (52.9)	53 489 (53.1)		
Year of index date				
2010–2013	3986 (19.8)	19.872 (19.8)	−0.009	0.437
2014–2017	5164 (25.6)	25.324 (25.1)		
2018–2021	6271 (31.1)	31 557 (31.3)		
2022–2024	4725 (23.5)	23 977 (23.8)		
Observation time prior to the index date (years), mean (SD)	8.6 (5.6)	8.6 (5.6)	−0.016	0.112
Diagnoses documented prior to index date				
Type 2 diabetes mellitus	5188 (25.8)	26 496 (26.3)	0.006	0.104
Obesity	2.349 (11.7)	12 083 (12.0)	0.003	0.180
COPD	2.649 (13.2)	12 769 (12.7)	−0.005	0.066
Nicotine dependence	1108 (5.5)	5.476 (5.4)	−0.001	0.717
Colorectal polyps	968 (4.8)	4.287 (4.3)	−0.005	0.001

Data are absolute samples and percentages unless otherwise specified.

COPD, chronic obstructive pulmonary disease; SMD, standardised mean difference.

### Association between autoimmune diseases and subsequent CRC diagnosis

As illustrated in table 2, the proportions of cases and controls diagnosed with each autoimmune disease are shown, in addition to the results of the logistic regression analysis. Patients with a first diagnosis of CRC did not exhibit a higher incidence of T1D compared with the control group, and T1D was not associated with CRC (OR 1.01; 95% CI 0.84 to 1.20). Conversely, the presence of IBD was significantly associated with the development of CRC (OR 1.53; 95% CI 1.33 to 1.75). No significant differences were observed between the CRC and control group for rheumatic diseases. Slightly positive associations between psoriasis and CRC (OR 1.11; 95% CI 0.97 to 1.27) and between Grave’s disease and CRC (OR 1.17; 95% CI 0.97 to 1.41) were observed, although they did not attain statistical significance. Autoimmune thyroiditis (OR 1.00; 95% CI 0.90 to 1.11) or multiple sclerosis (OR 0.89; 95% CI 0.68 to 1.17) was not associated with CRC. It is noteworthy that coeliac disease has also been observed not to be associated with the development of CRC (OR 1.06; 95% CI 0.69 to 1.64).

**Table 2:** Association between autoimmune disorders and a subsequent diagnosis of colorectal cancer in patients followed in general practices in Germany (all patients)

Variable	Proportion of among colorectal cancer cases (%)	Proportion among non-cancer controls (%)	OR (95% CI)	P value
Type 1 diabetes	0.74	0.73	1.01 (0.84 to 1.20)	0.957
Inflammatory bowel diseases	1.38	0.91	1.53 (1.33 to 1.75)	<0.001
Rheumatoid arthritis	2.53	2.63	0.94 (0.86 to 1.04)	0.246
Psoriasis	3.02	2.73	1.11 (0.97 to 1.27)	0.142
Systemic lupus erythematosus	0.04	0.04	0.91 (0.43 to 1.93)	0.801
Autoimmune thyroiditis	2.07	2.07	1.00 (0.90 to 1.11)	0.981
Graves’ disease	0.68	0.58	1.17 (0.97 to 1.41)	0.103
Multiple sclerosis	0.30	0.33	0.89 (0.68 to 1.17)	0.401
Coeliac disease	0.12	0.12	1.06 (0.69 to 1.64)	0.788
Ankylosing spondylitis	0.35	0.39	0.86 (0.67 to 1.12)	0.300

### Sex-stratified association between autoimmune diseases and subsequent CRC diagnosis

In view of the higher prevalence of autoimmune diseases in women, a sex-stratified analysis was performed, as illustrated in table 3. The presence of IBD was associated with a CRC in both women (OR 1.48; 95% CI 1.22 to 1.81) and men (OR 1.57; 95% CI 1.29 to 1.89). Similarly, a slight trend towards an increased association with CRC was observed in both female (OR 1.11; 95% CI 0.97 to 1.26) and male (OR 1.12; 95% CI 0.98 to 1.28) patients with psoriasis. The investigation revealed no statistically significant differences between male and female subjects in relation to the association between Graves’ disease and CRC.

**Table 3:** Association between sex, autoimmune disorders, and a subsequent colorectal cancer diagnosis among patients followed in general practices in Germany

Variable	Proportion among colorectal cancer cases (%)	Proportion among non-cancer controls (%)	OR (95% CI)	P value
Women				
Type 1 diabetes	0.49	0.61	0.80 (0.59 to 1.10)	0.176
Inflammatory bowel diseases	1.41	0.96	1.48 (1.22 to 1.81)	<0.001
Rheumatoid arthritis	3.46	3.54	0.96 (0.85 to 1.08)	0.489
Psoriasis	2.74	2.51	1.11 (0.97 to 1.26)	0.160
Systemic lupus erythematosus	0.07	0.07	0.97 (0.42 to 2.21)	0.934
Autoimmune thyroiditis	3.62	3.53	1.02 (0.91 to 1.15)	0.706
Graves’ disease	1.02	0.87	1.17 (0.93 to 1.47)	0.178
Multiple sclerosis	0.37	0.40	0.93 (0.65 to 1.34)	0.690
Coeliac disease	0.18	0.15	1.12 (0.65 to 1.94)	0.679
Ankylosing spondylitis	0.25	0.28	0.88 (0.57 to 1.37)	0.579
Men				
Type 1 diabetes	0.97	0.84	1.14 (0.92 to 1.42)	0.241
Inflammatory bowel diseases	1.35	0.87	1.57 (1.29 to 1.89)	<0.001
Rheumatoid arthritis	1.70	1.84	0.92 (0.78 to 1.08)	0.313
Psoriasis	3.27	2.93	1.12 (0.98 to 1.28)	0.157
Systemic lupus erythematosus	0.01	0.02	0.49 (0.06 to 3.84)	0.496
Autoimmune thyroiditis	0.70	0.78	0.89 (0.69 to 1.14)	0.358
Graves’ disease	0.37	0.32	1.17 (0.83 to 1.67)	0.366
Multiple sclerosis	0.23	0.28	0.82 (0.54 to 1.26)	0.374
Coeliac disease	0.08	0.09	0.90 (0.42 to 1.92)	0.790
Ankylosing spondylitis	0.43	0.50	0.86 (0.62 to 1.18)	0.339

## Discussion

In this study, we were able to show that only IBD, and not any other autoimmune disease, is associated with a subsequent CRC. It has been demonstrated that both sexes are equally affected by this association.

It is an established fact that chronic inflammation of the colon, a condition that arises, for example, in cases of IBD, significantly increases the risk of developing CRC. This phenomenon can be attributed to a multitude of interconnected biological processes. Prolonged inflammation leads to the release of reactive oxygen species and inflammatory cytokines, which can damage the DNA of cells in the colon lining.^14^ Over time, this DNA damage can lead to mutations that contribute to the development of cancer. Concurrently, inflammation has been demonstrated to interfere with the body’s intrinsic DNA repair systems, thereby facilitating the accumulation of these mutations. As the colon’s inner lining is subject to constant injury and subsequent repair, the body’s response is an escalation in cell turnover, thereby augmenting the probability of errors occurring during cell division.^17^ If key regulatory genes such as p53 are affected, abnormal cell growth can begin. The inflamed environment also becomes more conducive to cancer development.^18^ Inflammatory molecules promote the survival and growth of mutated cells, encourage the formation of new blood vessels to supply growing tumours and weaken the immune system’s ability to eliminate abnormal cells. It is evident that chronic inflammation has the capacity to modify the composition of the gut microbiome, thus resulting in an augmentation of harmful bacteria that may further promote cancer. In addition to genetic mutations, long-term inflammation can cause epigenetic changes that further contribute to the loss of tumour suppressive functions and the activation of cancer-promoting pathways.^19 20^ Taken together, this complex interplay of DNA damage, immune dysfunction, altered cell behaviour and microbial imbalance creates a high-risk environment for the development of CRC in individuals with chronic bowel inflammation.

Patients with IBD also exhibit systemic inflammation.^21^ In this condition, an abnormal immune response is triggered against intestinal microbes or self-antigens in genetically susceptible individuals. This results in the continuous activation of immune cells in the intestinal mucosa, including T cells, macrophages, dendritic cells, and neutrophils.^22 23^ These activated immune cells release a wide range of proinflammatory cytokines and chemokines, including tumour necrosis factor-alpha (TNF-α), interleukin-1β (IL-1β), IL-6, IL-17 and interferon-gamma.^24^ While these mediators are principally designed to control microbial threats in the gut, there is a possibility that they may enter the systemic circulation and affect distant organs and tissues. The persistent circulation of these inflammatory molecules contributes to a state of chronic low-grade systemic inflammation. This systemic inflammatory response is detectable in patients with IBD even during periods of clinical remission and is often reflected in elevated markers such as C-reactive protein and elevated levels of circulating cytokines.^25^ In addition, intestinal barrier dysfunction, a hallmark of IBD, allows the translocation of microbial products such as lipopolysaccharides into the bloodstream. This microbial translocation further stimulates systemic immune responses by activating toll-like receptors on immune cells in peripheral tissues.^26^ Systemic inflammation in IBD has clinical consequences beyond the gastrointestinal tract. It contributes to the development of extraintestinal manifestations such as arthritis, uveitis, skin disorders (eg, erythema nodosum), and hepatobiliary complications such as primary sclerosing cholangitis.^27^ Additionally, chronic systemic inflammation is associated with an increased risk of thromboembolic events, metabolic disorders, and possibly cardiovascular disease in patients with IBD.^28 29^

Systemic inflammation is also a hallmark of T1D, an autoimmune disease in which the immune system targets and destroys insulin-producing beta cells in the pancreas. This autoimmune attack triggers the release of pro-inflammatory cytokines (eg, TNF-α, IL-6, IL-1β) that circulate in the bloodstream and contribute to low-grade systemic inflammation.^30^ Chronic hyperglycaemia, a hallmark of T1D, further exacerbates inflammation through the formation of advanced glycation end-products, which activate immune cells and promote inflammatory pathways.^31^ The systemic inflammatory state in T1D is associated with an increased risk of complications, including cardiovascular disease, nephropathy, and retinopathy.^32^ We have shown that T1D is not associated with the development of CRC. This phenomenon may be attributed to the absence of migration of inflammatory cells in this condition to the colon, thereby preventing the propagation of inflammation to the bowel.

The migration of immune cells to the intestine is a highly regulated process involving signalling molecules and adhesion molecules that guide their movement from the bloodstream to the inflamed tissue. This process begins with the activation of immune cells, such as T cells and macrophages, in response to signals from the gut. Chemokines (eg, CCL25) and adhesion molecules (eg, α4β7 integrin) are expressed both on the endothelial cells of blood vessels and on the immune cells themselves. These molecules facilitate the adhesion of immune cells to the blood vessel walls in the mesenteric circulation and allow them to migrate across the endothelium into the intestinal tissue.^33^ In the context of IBD, the immune system is characterised by persistent activation that is dysregulated. The intestinal immune system mistakenly recognises normal gut microbiota or self-antigens as threats, leading to persistent inflammation. The specialised immune system of the gut, including the gut-associated lymphoid tissue, drives the recruitment of immune cells to the gut in response to these signals, contributing to the persistent inflammation and tissue damage characteristic of IBD.^34^ In contrast, other autoimmune diseases, such as T1D or RA, do not involve the same level of tissue-specific immune activation in the intestines. In these conditions, immune cells primarily target other organs, such as the pancreas in T1D or the joints in RA. Although systemic inflammation may occur, it does not trigger the specific molecular signals needed for immune cells to migrate to the intestine, thereby preventing inflammation from spreading to the gut. This may provide a rationale for the observation that, except for IBD, autoimmune diseases do not appear to be associated with the development of CRC. In contrast, some studies have shown that the occurrence of RA may reduce the risk of CRC.^35 36^ Possible explanations include the increased frequency of routine examinations, such as colonoscopies, among patients with RA, which may lead to earlier detection and a lower rate of CRC. It has also been discussed whether the systemic inflammation described above might not only have pro-tumourigenic effects but also anti-tumourigenic effects by enabling the activated immune system to eliminate emerging tumour cells more effectively. In our study, however, we did not observe a reduced risk. It is possible that treatment with Disease-Modifying Anti-Rheumatic Drugs(DMARD), such as JAK inhibitors, which have been shown to increase cancer risk, could counteract this potential protective effect.^37^ To investigate this further, detailed information on the ongoing therapies of patients would be necessary, but unfortunately, these data were not available to us. This hypothesis should be examined in future studies.

Our study has several limitations inherent to its database-based design. First, we cannot rule out the possibility of diagnostic misclassification or missing entries due to limitations within the ICD-10 coding system. Additionally, the German Disease Analyzer Database lacks information on patients’ lifestyle factors and socioeconomic status. The number of cases for conditions such as SLE and coeliac disease was too low to yield statistically robust conclusions. Furthermore, it should be noted that not all major autoimmune diseases were included in the study. For instance, atopic dermatitis is infrequently recorded by general practitioners. This limitation, however, impedes the capacity to draw sweeping conclusions about autoimmune diseases in their totality.

Another important limitation of our study is the residual imbalance in the prevalence of colorectal polyps between cases and controls, despite including this variable in the propensity score matching. Colorectal polyps are a well-established precursor to CRC, and their slightly higher prevalence in the cancer group (4.8%) compared with controls (4.3%) may have introduced residual confounding. Achieving perfect matching on multiple risk factors, including polyps, is inherently challenging in large-scale observational datasets. However, the absolute difference between groups is small and is unlikely to have materially influenced the results of our regression models. Nonetheless, we acknowledge this as a relevant limitation.

Nevertheless, the database provides valuable insights into general practitioner consultations in Germany. The findings of this study demonstrate that, with the exception of IBD, the autoimmune diseases are not associated with CRC. This further underscores the significance of periodic screening for patients with IBD in the future.

## Supplementary material

10.1136/bmjgast-2025-001886 online supplemental file 1

## Data Availability

Data may be obtained from a third party and are not publicly available.
